# Editorial commentary: public mental health and racism in Europe

**DOI:** 10.1186/s13690-021-00722-0

**Published:** 2021-11-17

**Authors:** Patrick Cloos, Johan Bilsen

**Affiliations:** 1 School of Public Health and School of Social Work, University of Montreal, Québec, Canada; 2 Department of Public Health, Mental Health and Wellbeing research group, Vrije Universiteit Brussel, Ixelles, Belgium

Several dramatic events, related to racism, have made headlines globally in recent months. The murder of George Floyd by a police officer in the United States is a tragedy that reinforced the Black Lives Matter movement which fights against police violence inflicted against Black people in the United States. The global media coverage of both the murder and the anti-racist protest movements have contributed to raising awareness not only of police violence but also the persistence of racism. In Europe, racial profiling and increasing violence in everyday interactions, during police checks or in detention of Afro-descendants in particular, whether they are European citizens or migrants, have been denounced by the European parliament [1]. Those who are designated ‘Muslim’ or ‘Black’ are particular victims of racism, as it was noticed in Belgium, whether in terms of negative attitudes towards them (insults, threats, aggression) or unemployment [2]. Occupational downgrading and unemployment, as for Afro-descendants, contrast with a generally high level of education. In a recent study on Afro-descendants in Belgium, Demart et al. indicated that a majority of respondents (80%) declared experiences of discrimination, unequal treatment or insults with references to skin colour or origin [3].

Racism is a type of discrimination with a physical motive. According to M. Foucault [4], European States used racism as a power to justify slavery and modern colonialisms (among which are Belgium, France, Germany, Spain, Italy, The Netherlands, and Spain). Racism aims at both constructing and producing *difference* targeting bodies (racialization), while establishing a hierarchical relationship between the *self* (viewed as the norm) and the *racialized others* (viewed as *different*), and hence the mistreatment of the later. Foucault viewed racism as a condition for the legitimising of the killing of those categorized as inferior.

With regard to health, racism is seen as a social determinant of health and a global public health issue [5] that also affects mental health [6, 7]. Racism determines social inequalities in health, which are amplified in the context of disastrous situations. For example the current COVID-19 pandemic [5], through discriminatory health policies that can aggravate precariousness, or through acts of violence [8].

Authors note the importance of research on the deleterious impact of racism on health, including the lack of attention regarding relationships between the macro-micro levels of racism [9]. Racism has structural and institutional components that shape social hierarchy and daily interactions, resulting in potential social exclusion and deleterious consequences for racialized minorities [9]. Importantly, racism also interacts with other structural determinants (e.g., migration law and migrant status, class and gender power relations) that shape social position and that determine living and working circumstances, and overall well-being [10].

With regard to mental health, racism can be a normative experience for some minorities, resulting in psychological distress and poor mental health [7]. Chronic exposure to racism worsens mental health [11]. Racism has social and economic consequences, which in turn impact negatively on mental health of racialized minorities. Furthermore, racism negatively affects the mental health system and appropriate and timely mental health services of these groups [12]. Research reports how quality of mental health support to ethnic minority women depends on cultural competence and attitudes of health care providers [13]. Racialized stereotypes even affect the diagnostic process [14]. Also the health of youth is affected by racism, and it is suggested that psychosocial resources can improve some of its adverse effects [15].

The European parliament acknowledges the problem, and urges the EU and national authorities to develop anti-racist policies and end racism, e.g. in education, housing, healthcare, police, political participation and migration [1]. Public health research on institutionalized racism can contribute to overcoming its deleterious effects on mental health [16, 17]. It must focus on the relationship between policies, social position, living and working conditions, access to health care and other resources, and psychosocial indicators (e.g., stress), and mental health outcomes. It must also contribute in dismantling systems of oppression such as racism [8]. Public health, as an institution, should exercise leadership in this area by putting in place internal policies and practices for change and more social justice. This could be operationalised through more inclusive policies towards historically excluded and marginalised populations, by encouraging for example the recruitment of people (researchers, students and other health professionals) who identify themselves as racialized minorities. Also other proactive policies are necessary such as a call to the health and research community to train and sensitize mental health professionals against racism, review policies and regulations, and collect data on racialized and ethnic categories for public health surveillance purposes.

## Data Availability

Not applicable.
